# Afternoon exercise is more efficacious than morning exercise at improving blood glucose levels in individuals with type 2 diabetes: a randomised crossover trial

**DOI:** 10.1007/s00125-018-4767-z

**Published:** 2018-11-13

**Authors:** Mladen Savikj, Brendan M. Gabriel, Petter S. Alm, Jonathon Smith, Kenneth Caidahl, Marie Björnholm, Tomas Fritz, Anna Krook, Juleen R. Zierath, Harriet Wallberg-Henriksson

**Affiliations:** 1 Department of Physiology and Pharmacology, Section of Integrative Physiology, Karolinska Institutet, Solnavägen 9, Biomedicum (C4), 171 77 Stockholm, Sweden; 2 Faculty of Medicine, University of Oslo, Oslo, Norway; 3 Department of Molecular Medicine and Surgery, Section of Integrative Physiology, Karolinska Institutet, Stockholm, Sweden; 4 Department of Clinical Physiology, Karolinska University Hospital, Stockholm, Sweden; 5 The Novo Nordisk Foundation Center for Basic Metabolic Research, Faculty of Health and Medical Sciences, University of Copenhagen, Copenhagen, Denmark

**Keywords:** Blood glucose level, Circadian rhythm, Continuous glucose monitoring, Exercise, High-intensity interval training, Type 2 diabetes

## Abstract

**Aims/hypothesis:**

Exercise is recommended for the treatment and prevention of type 2 diabetes. However, the most effective time of day to achieve beneficial effects on health remains unknown. We aimed to determine whether exercise training at two distinct times of day would have differing effects on 24 h blood glucose levels in men with type 2 diabetes.

**Methods:**

Eleven men with type 2 diabetes underwent a randomised crossover trial. Inclusion criteria were 45–68 years of age and BMI between 23 and 33 kg/m^2^. Exclusion criteria were insulin treatment and presence of another systemic illness. Researchers were not blinded to the group assignment. The trial involved 2 weeks of either morning or afternoon high-intensity interval training (HIIT) (three sessions/week), followed by a 2 week wash-out period and a subsequent period of the opposite training regimen. Continuous glucose monitor (CGM)-based data were obtained.

**Results:**

Morning HIIT increased CGM-based glucose concentration (6.9 ± 0.4 mmol/l; mean ± SEM for the exercise days during week 1) compared with either the pre-training period (6.4 ± 0.3 mmol/l) or afternoon HIIT (6.2 ± 0.3 mmol/l for the exercise days during week 1). Conversely, afternoon HIIT reduced the CGM-based glucose concentration compared with either the pre-training period or morning HIIT. Afternoon HIIT was associated with elevated thyroid-stimulating hormone (TSH; 1.9 ± 0.2 mU/l) and reduced T_4_ (15.8 ± 0.7 pmol/l) concentrations compared with pre-training (1.4 ± 0.2 mU/l for TSH; 16.8 ± 0.6 pmol/l for T_4_). TSH was also elevated after morning HIIT (1.7 ± 0.2 mU/l), whereas T_4_ concentrations were unaltered.

**Conclusions/interpretation:**

Afternoon HIIT was more efficacious than morning HIIT at improving blood glucose in men with type 2 diabetes. Strikingly, morning HIIT had an acute, deleterious effect, increasing blood glucose. However, studies of longer training regimens are warranted to establish the persistence of this adverse effect. Our data highlight the importance of optimising the timing of exercise when prescribing it as treatment for type 2 diabetes.

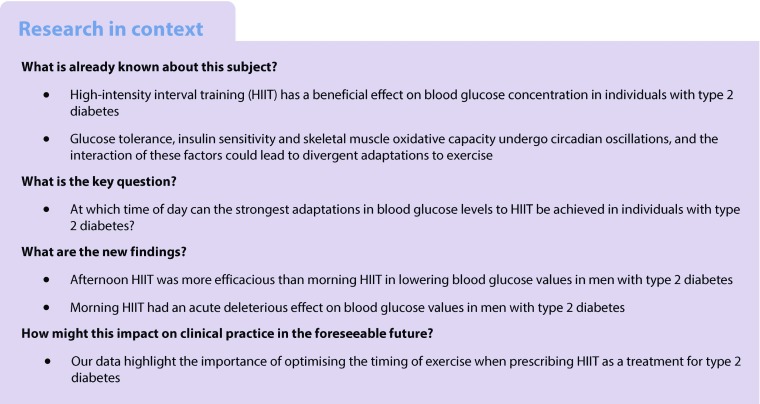



## Introduction

Exercise is recommended for the prevention and treatment of type 2 diabetes [1]. Low-volume high-intensity interval training (HIIT) remodels skeletal muscle and the cardiorespiratory system to a similar extent as continuous moderate-intensity training, with reduced time commitment and exercise volume [2]. In addition, glucose tolerance, insulin sensitivity and muscle oxidative capacity show circadian oscillations [3, 4]. Interactions between these factors could lead to divergent physiological adaptations to exercise training at different times of day. By determining the effects of morning and afternoon HIIT on 24 h blood glucose in men with type 2 diabetes in ‘free-living’ conditions, we tested the hypothesis that time of day would influence the metabolic response to exercise.

## Methods

This trial was approved by the regional ethics board at the Karolinska Institutet, Stockholm, Sweden (2016/1421-31/1). All participants gave their informed consent.

### Study design

Eleven men with type 2 diabetes (Table 1) completed a randomised crossover trial. The trial was intended as a pilot to a larger-scale study (ClinicalTrials.gov number NCT03553524). Inclusion criteria were age between 45 and 68 years and a BMI between 23 and 33 kg/m^2^. Exclusion criteria were insulin treatment, recent history of smoking (<6 months) and presence of cardiovascular, rheumatoid, blood-borne or neoplastic disease. Participants were receiving either dietary treatment (*n* = 2) or metformin (*n* = 9; daily dose range 500–3000 mg; *n* = 2, 3/day; *n* = 4, 2/day; *n* = 3, 1/day) and continued their treatment throughout the study. Blood samples were obtained before and after completion of each training regimen (Table 2). Participants kept dietary records for 24 h before blood sampling and were asked to replicate their diet prior to subsequent visits.

**Table 1:** Baseline characteristics

Variable (*n* = 11)	Baseline data
Age (years)	60 ± 2
Time since diagnosis (years)	11 ± 3
BMI (kg/m^2^)	27.5 ± 0.6
WHR	0.99 ± 0.01
Body fat (%)	26.4 ± 0.8
Max heart rate (beats/min)	158 ± 7
Maximum power (W/kg)	2.1 ± 0.1

Data are mean ± SEM

**Table 2:** Blood chemistry analyses

Blood chemistry	Pre-training	Post-morning	Post-afternoon
Glucose (mmol/l)	7.3 ± 0.3	7.7 ± 0.4	7.5 ± 0.3
Insulin (pmol/l)	56.9 ± 9.3	71.4 ± 6.9	70.4 ± 11.6
HbA_1c_ (mmol/mol)	48.3 ± 3.9	45.1 ± 2.1	46.1 ± 2.7
HbA_1c_ (%)	6.6 ± 0.4	6.3 ± 0.2	6.4 ± 0.2
Total cholesterol (mmol/l)	4.2 ± 0.4	4.4 ± 0.3	4.2 ± 0.4
HDL-cholesterol (mmol/l)	1.2 ± 0.1	1.3 ± 0.1	1.2 ± 0.1
LDL-cholesterol (mmol/l)	2.4 ± 0.4	2.4 ± 0.4	2.3 ± 0.4
Triacylglycerol (mmol/l)	1.2 ± 0.2	1.6 ± 0.3	1.4 ± 0.2
PTH (pmol/l)^††^	3.9 ± 0.2	4.4 ± 0.2	4.6 ± 0.3^‡^
TSH (mU/l)^††^	1.4 ± 0.2	1.7 ± 0.2^‡^	1.9 ± 0.2^‡^
T_4_ (pmol/l)^†^	16.8 ± 0.6	16.1 ± 0.7	15.8 ± 0.7^‡‡^
T_3_ (pmol/l)	4.7 ± 0.2	4.8 ± 0.2	4.9 ± 0.1

Blood samples were obtained from fasting participants before (Pre-training) and after HIIT training for 2 weeks in the morning (Post-morning) or afternoon (Post-afternoon). Data are mean ± SEM. One-way ANOVA (^†^*p* < 0.1, ^††^*p* < 0.05), followed by Tukey’s post hoc test (^‡^*p* < 0.1, ^‡‡^*p* < 0.05 vs pre-training)

Continuous glucose monitors (CGMs; FreeStyle Libre, Abbott Laboratories, Chicago, IL, US) were fitted, and baseline (pre-training) measurements were collected for 2 weeks. Participants were assigned (block randomisation, size = 4 or 5) to either morning (08:00 hours) or afternoon (16:00 hours) training (*n* = 7 and *n* = 4, respectively) for 2 weeks; this consisted of supervised exercise sessions on Mondays, Wednesdays and Fridays (Exercise), followed by a rest day (Rest) (giving six exercise sessions in total). Following a 2 week ‘wash-out’ period, participants performed the opposite exercise training regimen. Two participants who completed only three and four afternoon sessions, respectively, were excluded from the week 2 comparisons. Researchers were not blinded to the group assignment.

### Exercise intervention

Supervised exercise training was performed at a gym in Stockholm. At a pilot test, each individual cycled at 75 rpm and with a load that forced a rest after 1 min. This was repeated six times with a 1 min rest in between. The load reached for each 1 min pulse was recorded, and a mean of the six pulses was used for all HIIT sessions. HIIT sessions were performed on a cycle ergometer, commencing with a 7 min warm-up, followed by six 1 min pulses at the predetermined load (usually above 220 W, range 180–350 W) and with a pedalling rate of 75 rpm. Each pulse was followed by a 1 min recovery period (75 rpm with minimal load). Participants were instructed to have a light breakfast before (approximately 1 h ahead of) the morning sessions, and a standardised snack (sandwich and juice) was offered ad libitum around 30 min after the exercise. Afternoon sessions took place after lunch (approximately 3 h later) and were followed by dinner around 30 min thereafter.

### Statistical analysis

Blood glucose level was determined using CGMs throughout the study, from which hourly means were calculated. The 24 h blood glucose measurements for the first three (week 1) and the last three (week 2) Exercise and Rest days for each trial were compared with each other and with corresponding pre-training days. Pre-training days were matched to corresponding days of the week for Exercise and Rest, respectively. Data were analysed using Prism 7.01 software (GraphPad, San Diego, CA, US) by two-way ANOVA followed by a Tukey’s post hoc test (significance level set at *p* < 0.05).

## Results

**Fig. 1:**
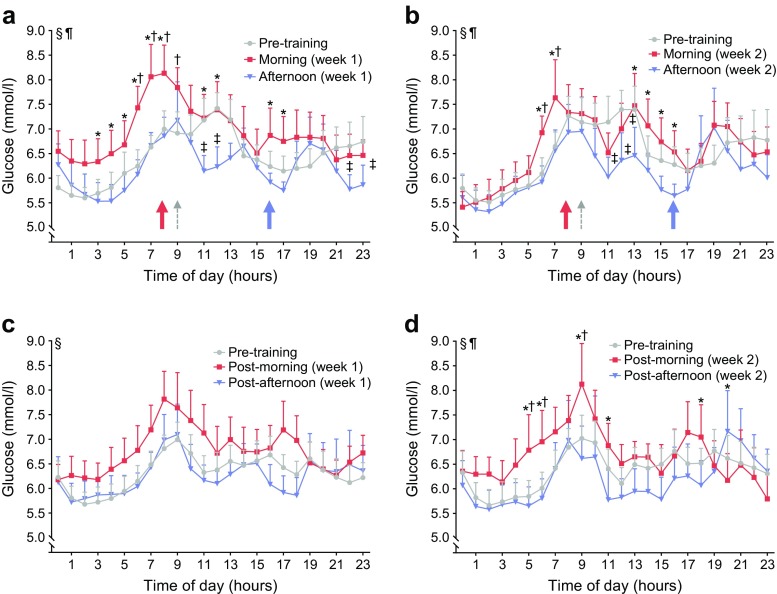
CGM-based glucose levels in response to HIIT. CGM-based glucose levels were assessed during the pre-training period and on exercise days (Exercise) and subsequent days (Rest). Blood glucose readings on Exercise days in (**a**) week 1 (*n* = 11) and (**b**) week 2 (*n* = 9), and on Rest days in (**c**) week 1 (*n* = 11) and (**d**) week 2 (*n* = 8). Red lines and symbols, morning exercise; blue lines and symbols, afternoon exercise; grey lines and symbols, matched pre-training days. Red arrows, time of morning exercise; blue arrows, time of afternoon exercise; grey arrows, snack offered. Using two-way ANOVA: ^§^*p* < 0.05 for the effect of time; ^¶^*p* < 0.05 for the interaction between exercise and time. Using Tukey’s multiple comparison test: *p < 0.05 for the difference between exercise trials; ^†^*p* < 0.05 for the difference between morning exercise trial and pre-training period; ^‡^*p* < 0.05 for the difference between afternoon exercise trial and pre-training period. Values are means + SEM

Morning HIIT increased CGM-based glucose concentrations on Exercise days during week 1 (6.9 ± 0.4 mmol/l; mean ± SEM) (Fig. 1a) and week 2 (6.6 ± 0.4 mmol/l) (Fig. 1b) compared with either the pre-training days (6.4 ± 0.3 mmol/l) or afternoon HIIT (6.2 ± 0.3 and 6.1 ± 0.4 mmol/l for week 1 and 2, respectively) at several time points. Conversely, afternoon HIIT reduced CGM-based glucose concentration on Exercise days during week 1 (Fig. 1a) and week 2 (Fig. 1b) of training compared with either pre-training or morning HIIT at several time points. Morning HIIT also increased CGM-based glucose concentrations on Rest days during week 2 (Fig. 1d), but not week 1 (Fig. 1c), compared with pre-training days or afternoon HIIT at several time points. Mean 24 h glucose curves for the pre-training period and the last 3 days of the wash-out period were unaltered (data not shown). Thus, the wash-out period was sufficient to remove the effects of HIIT. Afternoon HIIT elevated parathyroid hormone (PTH) and thyroid-stimulating hormone (TSH) concentrations, and reduced T_4_ concentration, compared with pre-training concentrations (Table 2). TSH was also elevated after morning HIIT, whereas PTH and T_4_ concentrations were unaltered.

## Discussion

To our knowledge, this is the first study performed in a real-life setting that compares the impact of 2 weeks of HIIT at different times of day on blood glucose excursions in people with type 2 diabetes. Afternoon HIIT was more efficacious than morning HIIT in improving blood glucose levels. Unexpectedly, morning HIIT elevated glucose levels at several time points throughout the day compared with the pre-training period and afternoon HIIT. Conversely, afternoon HIIT lowered blood glucose at several time points throughout the day compared with pre-training and morning HIIT. Our results are clinically relevant, showing that timing of exercise affects the glycaemic response to HIIT in men with type 2 diabetes.

The circadian clock is a key homeostatic regulator that is synchronised by photic and non-photic stimuli (food intake, temperature and physical activity), and controls many genomic and physiological responses in virtually all cells [1]. We reasoned that if the timing of exercise could be optimised to coincide with these responses, its potency as a therapeutic tool might be augmented. Several lines of evidence suggest that the timing of exercise alters physiological responses diversely. In healthy young men, parasympathetic and sympathetic activity during the next nocturnal sleep is differentially enhanced by morning and evening exercise, as evidenced by changes in body temperature, heart rate and heart rate variability [5]. Moreover, human skeletal muscular strength and mitochondrial function peak in late afternoon [4, 6], suggesting a circadian rhythm of oxidative metabolism.

Here we report that the time of day affects the blood glucose response to exercise in people with type 2 diabetes, with improvements in daily glucose excursions observed in conjunction with afternoon HIIT. Our results are compatible with a previous report that a session of moderate-intensity exercise performed in the morning vs the afternoon conferred a lower risk of late-onset hypoglycaemia and improved metabolic control on the subsequent day in young individuals with type 1 diabetes [7]. We report that morning HIIT increased CGM-based glucose concentration acutely as well as on the subsequent rest day during week 2 of training. Collectively, these results imply that the timing of exercise impacts blood glucose excursions over the day. Aligning exercise bouts with the systemic circadian rhythm appears to confer a differential glycaemic response in people with diabetes.

Optimising the timing of an exercise session with defined eating patterns may interact with the circadian clock signal to prevent disease pathogenesis [1]. Morning HIIT has been shown to improve blood glucose levels in individuals with type 2 diabetes when performed under controlled conditions with standardised meals provided 1.5 h before the exercise session [8]. In our study, morning HIIT had an acute deleterious effect on blood glucose. Conceivably, individuals participating in an exercise programme in a real-life setting may consume meals closer to or directly after the morning exercise session. Such a scenario, potentiated by the suppressive effects of high-intensity exercise on gastric emptying [9], may partially account for deleterious effects of morning HIIT on blood glucose in our study. Thus, timing is also important in respect to food intake, with a better glycaemic response achieved with postprandial exercise in people with type 2 diabetes [10].

Exercise at different times of day may coincide with fuel utilisation preferences on the part of the working muscles. Moreover, HIIT has a preference for carbohydrate oxidation when compared with moderate-intensity exercise [11]. Thus, differential fuel utilisation patterns during morning vs afternoon exercise may partly explain the changes in blood glucose regulation between trials. Morning HIIT-induced increases in blood glucose levels were predominately observed in week 1. An acute dysregulation of diurnal hormonal rhythms could account for this phenomenon. Supporting this assertion, afternoon HIIT potentiated the TSH and T_4_ responses. In week 2 of the morning HIIT, we observed an apparent improvement in glucose levels, as the participants become better acclimated to the training regimen. Studies of longer training periods are thus warranted to establish the persistence of these adverse effects.

We conducted a field-based study in ‘free-living’ individuals, so specific factors responsible for differing blood glucose levels between morning and afternoon HIIT trials remain to be elucidated. Due to the randomised crossover design and paired nature of the data, putative individual differences in the sleep/wake cycle and sleep architecture are mostly accounted for. Future studies are warranted to characterise the underlying hormonal and metabolic changes to exercise training at different times of day in healthy and diseased populations.

Although HIIT is promising for the management of type 2 diabetes, our data indicate that the timing of exercise should be considered. This concern may be modality and population dependent. In light of diverse hormonal responses and fuel utilisation preference [11], less intense forms of exercise may have differing effects on blood glucose and associated metabolic responses. Conversely, individuals prone to nocturnal hypoglycaemia or who are receiving insulin treatment might respond differently to the same training regimen. In conclusion, our data highlight the importance of optimising the timing of exercise sessions to improve glycaemic control in people with type 2 diabetes.

## Abbreviations

CGM: Continuous glucose monitor
HIIT: High-intensity interval training
PTH: Parathyroid hormone
TSH: Thyroid-stimulating hormone
